# Sexual and Reproductive Health Care for Irregular Migrant Women: A Meta-Synthesis of Qualitative Data

**DOI:** 10.3390/healthcare11111659

**Published:** 2023-06-05

**Authors:** José Granero-Molina, Ariadna Sara Gómez-Vinuesa, Gonzalo Granero-Heredia, Alba Fernández-Férez, María Dolores Ruiz-Fernández, Isabel María Fernández-Medina, María del Mar Jiménez-Lasserrotte

**Affiliations:** 1 Nursing, Phisiotheraphy and Medicine Department, University of Almería, 04120 Almería, Spain; mrf757@ual.es (M.D.R.-F.); isabel_medina@ual.es (I.M.F.-M.); mjl095@ual.es (M.d.M.J.-L.); 2 Associate Researcher, Faculty of Health Sciences, Universidad Autónoma de Chile, Santiago 7500000, Chile; 3 Nursing Unit, Andalusian Health Service, 04009 Almería, Spain; ariadna.gomez.sspa@juntadeandalucia.es (A.S.G.-V.); alba.fernandez.ferez.sspa@juntadeandalucia.es (A.F.-F.); 4 Physiotherapist Unit, Andalusian Health Service, 04009 Almería, Spain; ggh736@inlumine.ual.es

**Keywords:** irregular migrant women, meta-synthesis, qualitative research, public health

## Abstract

Migratory movements are a political, social, and public health issue on a global scale. Access to sexual and reproductive health services for irregular migrant women (IMW) is a public health issue. The aim of this study is to identify qualitative evidence of IMW’s experiences of sexual and reproductive health care in emergency and primary care settings. The methods employed involve conducting a meta-synthesis of qualitative studies. Synthesis includes assembling and categorising findings based on similarity in meaning. The search was conducted between January 2010 and June 2022 using PubMed, WOS, CINAHL, SCOPUS, and SCIELO databases. Out of the initial pool of 142 articles identified, only 9 fulfilled the predetermined criteria and were subsequently included in the review. Four main themes were established: (1) the need to focus emergency care on sexual and reproductive health; (2) unsatisfactory clinical experiences; (3) forced reproduction; and (4) alternating between formal and informal healthcare services. The attitudes of IMW towards sexual and reproductive health are influenced by culture, educational level, fear, barriers, and the attitude of healthcare providers. Healthcare institutions need to be aware of the IMW’s experiences to understand the specific difficulties they face. IMW calls for socially and culturally sensitive health care, cultural mediators, improved communication, and safe environments that ensure confidentiality.

## 1. Introduction

Migratory movements are a major political, social, and public health issue on a global scale [1]. The United Nations (UN) defines “international migrant” as a person who has changed their country of habitual residence, distinguishing between short-term migrants (3 months–1 year) and long-term migrants (≥ 1 year). According to the International Organization for Migration (IOM), the number of international migrants in 2019 reached 271.6 million people (3.5% of the world’s population), of whom 47.9% were women [2]. Europe has traditionally been a destination for international migrants; in 2019, it received 82.3 million, 51.4% of whom were women [2]. Almost 87 million international migrants were living in Europe in 2020, 16% more than in 2015 [3]. Spain ranked tenth among destinations for international migrants in 2020 with 5.5 million people [4], of whom 2.2 million were men and 2.3 million were women [5].

The term “irregular migration” refers to the movement of people outside the laws, regulations, or international agreements governing entry into or exit from the country of origin, transit, or destination [6]. Irregular migrants (IMs) enter a country without legal recourse, documentation, or refugee status and are not authorised to remain [7]. The term “irregular migrant women” (IMW) refers to a woman who, owing to unauthorised entry, breach of a condition of entry, or the expiry of her visa, lacks legal status in a transit or host country [6]. North Africa is a major transit hub and departure point for IMs from the Maghreb and sub-Saharan Africa heading to Europe. Tens of thousands of migrants attempt to reach Europe from North Africa via the Mediterranean routes every year [3], fleeing political persecution, armed conflict, climate change, or seeking better life opportunities. 

European countries must address the health needs of IMs. In Spain, the approval of Royal Decree-Law 16/2012 introduced restrictions on IMs’ access to health services [8]. However, the subsequent approval of Royal Decree-Law 7/2018 [9] guaranteed all people in Spanish territory the right to health care, regardless of their administrative status. The number of women of foreign origin in Spain has tripled since the beginning of the century, especially women of childbearing age [5]. In 2017, two out of ten children born in Spain were born to foreign mothers (mainly African and especially Moroccan). Foreign women accounted for 31% of hospitalisations for childbirth in 2018, as well as hospitalisations of children. Providing care to IMs is a challenge for receiving countries, where women and children in particular may have limited access to healthcare services [10]. Although they can access contraception, IMW are younger and have more children, so they use pregnancy, childbirth, and childcare services more frequently [5].

Sexual and reproductive health (SRH) encompasses physical, mental, and social well-being. SRH implies a person’s right to a healthy body, education, and the freedom to make their own decisions regarding sexual relations, pregnancy, abortion, sexually transmitted infections, and all forms of sexual violence/coercion [11]. Sexual and reproductive health care includes prenatal, perinatal, postpartum, and neonatal care; family planning; stopping unsafe abortions; preventing and treating sexually transmitted diseases, reproductive tract infections, and gynaecological conditions; and promoting healthy sexuality [12].

SRH is still the leading cause of death and suffering in women of childbearing age in humanitarian settings around the world. IMW are often in greater need of SRH services yet have limited access to them. Furthermore, they are often overlooked and are at increased risk of morbidity and mortality [13]. IMW experience high-risk sexual encounters and exploitation at various stages of the migration process. IMW are more exposed to sexually transmitted diseases, sexual violence, rape, unwanted pregnancies, and unsafe abortions [14]. Providing IMW with access to sexual and reproductive health (SRH) services has become a major public health objective. Several studies have examined this problem on an epidemiological, socioeconomic, or clinical level [1,15,16]. Understanding the experiences of physicians [17], nurses [18], and healthcare providers in caring for IMW could be important for removing barriers, introducing improvements, and developing specific protocols. Likewise, gaining insight into the experiences of accompanied and unaccompanied minors [19], as well as those of IMW, in accessing emergency and primary care services is key to improving their care. Although several studies focus on the experiences of IMW [20,21], a synthesis of aggregated data is needed to gain a deeper understanding of the phenomenon [22] in order to guide clinical practice and provide quality care to these women. The research question guiding this review is: What are the experiences of IMW with regard to SRH care in emergency and primary care? The aim of the study is to identify qualitative evidence of IMW’s experiences of SRH care in emergency and primary care settings.

## 2. Materials and Methods

### 2.1. Design

This is a systematic review and meta-synthesis of qualitative studies. The meta-synthesis involves assembling and categorising findings based on the similarity of meaning, resulting in a set of statements that consolidate and reflect on knowledge in the field. This review follows the ENTREQ (Enhancing Transparency in Reporting the Synthesis of Qualitative Research) guidelines [23].

### 2.2. Search Methods

PubMed, WOS, CINAHL, SCOPUS, and SCIELO databases were searched for qualitative studies in English and Spanish published between 2010 and January 2023 (Table 1). The SPIDER method was used for qualitative research (sample, phenomenon of interest, design, evaluation, and type of research) [24].

### 2.3. Inclusion and Exclusion Criteria

Inclusion criteria: IMW who have experienced SRH care in emergency and primary care (prenatal, perinatal, postpartum, and neonatal care); family planning; abortions; sexually transmitted diseases, reproductive tract infections, or gynaecological conditions; qualitative research or mixed methodology articles (phenomenology, ethnography, grounded theory, etc.) were considered. Only complete original research articles published in English or Spanish between 2010 and 2023 were included. Exclusion criteria: non-primary articles, editorials, opinion pieces, or abstracts were excluded from the study.

### 2.4. Results of the Search

**Figure 1:**
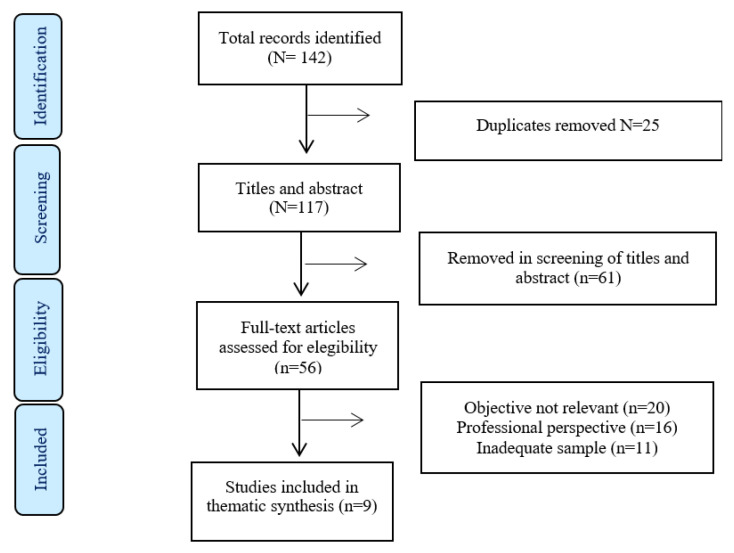
Flowchart.

A five-stage selection process was performed: duplicate elimination, title selection, abstract review, full paper review, and reference tracking. A total of 142 studies were found; nine articles fulfilled the inclusion criteria and were included in this review (Figure 1).

### 2.5. Quality Assessment

Each primary study was assessed using the Joanna Briggs Institute’s Qualitative Assessment Rating Instrument (QARI) [25]. The included articles were considered to be of high quality based on their objectives, design, analysis, results, and useful insights into the topic (Table 2). No studies were excluded as a result of the quality assessment. All studies demonstrated clear objectives and appropriate research.

### 2.6. Data Extraction

All duplicate records were removed. The researchers analysed the selected studies by extracting data on the author, year, country, design, sample, age, and research topic. It was not necessary to contact the authors of the primary studies to obtain recommendations. The references to the included papers were reviewed.

#### Data Synthesis and Analysis

The included studies were analysed thematically. The synthesis was undertaken by AGV and verified by JGM. Two independent reviewers with expertise in IMW and qualitative research verified the results. The thematic synthesis of qualitative data (Table 3) included line-by-line coding, developing descriptive themes, and generating themes and sub-themes in three stages [30]:

### 2.7. Rigor

To assess the validity of the review, we kept structured summaries of each original study. We checked whether emerging findings were transferable to different study contexts, and we tried to distinguish between primary care and emergency care participants. In addition, we looked to see if our synthesis findings could be attributed to a particular group of IMW. Following the thematic synthesis, we examined the studies’ contributions to the final analytical themes and recommendations for intervention.

## 3. Results

The nine qualitative studies comprised a total sample of 179 IMW from Cambodia, Nigeria, Bolivia, Ecuador, Portugal, Brazil, Bangladesh, Dominican Republic, Nigeria, Morocco, Spain, Macedonia, Romania, Bosnia, Albania, Somalia, Afghanistan, Ethiopia, and Mexico, aged between 18 and 40 (Table 4). Thematic synthesis is an inductive process in which four themes and nine sub-themes emerge (Table 5).

### 3.1. The Need to Focus Emergency Care on SRH

Migratory travel under adverse conditions increases the vulnerability of IMW, who can often be victims of physical, psychological, and sexual violence. It is necessary to implement emergency care protocols focused on these women’s SRH and on the physical and psychological traumas they suffer. Healthcare professionals need to know how to intervene and therefore require specific training on SRH.

#### 3.1.1. IMW: Victims of Trafficking and Sexual Exploitation

During the migration process, IMW are handed over to human trafficking networks that use them as bargaining chips for border crossings, bribes, and exchanges. When IMW arrive in the country of destination with physical, psychological, or social problems, the emergency care they are provided should specifically cover SRH and safety. This care not only includes physical, obstetric, and gynaecological assessments but also the recognition of scars, bruises, or tattoos indicative of violence and trafficking. As one participant says, specific screening is needed to address their physical health and to ascertain whether they are victims of human trafficking or covert prostitution.

*“They come and rape you for days, and when it suits them, they leave you there, bleeding... and you have to continue your journey as best as you can”*

[10]

#### 3.1.2. The Need to Develop Suitable Safety Protocols

The migration journey has consequences for IMW that need to be understood by healthcare providers. IMW are often distrustful and depressed, with fragile and unpredictable states of mind. Alongside the health care they are provided, IMW also need reassurance, respect, and time, which means developing culturally adapted care protocols. IMW arrive with concerns for their health and that of their children, as well as for the families they have left behind in Africa. The mafia often prostitutes IMW on their arrival in Europe by coercing them with threats to their families. IMW need healthcare protocols to be implemented that focus on both SRH and their safety.

*“Who is protecting my baby?” “Who is protecting my family?”*

[10]

IMW are particularly exposed during all phases of the migration process and reach emergency care feeling exhausted, fearful, and unsafe. They may have suffered rape, genital mutilation, backstreet abortions, or received informal care for SRH problems, thus increasing their risk of having health issues. The prevention and follow-up of STIs should also be emphasised as a key issue to be taken into account in primary healthcare settings.

### 3.2. Unsatisfactory Clinical Experiences

The clinical studies on healthcare providers reveal a lack of awareness and training when dealing with or interacting with IMW. These women are scared, have little information, and are unaware of their rights. They perceive that healthcare providers are in a rush. An Eritrean IMW in Switzerland spoke of her loneliness and lack of social support from family members:

*“When you have your parents with you, they are there to help because they feel responsible for you.” Consequently, I find it harder in Switzerland compared to Eritrea”*

[21]

This is compounded by the fact that they are not always attended to by the same professionals, leading them to feel uncomfortable and insecure. This is how a Bolivian woman described it:

*“I think it is never the same doctor from one ultrasound to the other.” “That is difficult.”*

[21]

#### 3.2.1. The Need for Interpreters

Most IMW do not speak the language of the host country, which hinders communication and the clinical assessment of SRH. This communication barrier, along with a lack of information, heightens IMW’s perception of being vulnerable and unable to access care. They sometimes referred to a lack of control, a fear of not being understood, and an inability to understand healthcare professionals’ recommendations. IMW’s perception of being misled and ignored leads them to feel distrustful and unsafe. They believe that healthcare professionals treat them differently from others, which worsens the therapeutic relationship.

*“I rang the bell several times, asking for help.” “I was worried that something was wrong with the baby, who was screaming and screaming.” “After a long time, the staff came in and said something incomprehensible in Swedish, then they left and did not come back.”*

[27]

The inadequate explanation of medical procedures that IMW undergo, such as the first-trimester risk assessment for trisomy, leads to feelings of confusion, fear, misunderstanding, anxiety, and even denial. This is how one IMW explains it:

*“She (the physician) did not explain what the test would be like properly; I thought it was the one with the needle, so I said no.”*

[21]

#### 3.2.2. Healthcare Providers’ Lack of Cultural Competence

IMW can feel rejected when attending emergency or primary care services. Healthcare providers are often cagey and unempathetic, which leads IMW to feel insecure. Some healthcare professionals expressed concerns about the undocumented status of IMW, reflecting their lack of cultural competence in providing care. This leads to feelings of fear, rejection, and discrimination. IMW feel that healthcare professionals normalise significant health conditions they present, do not listen to them, and doubt what they are telling them, which makes them more concerned about their situation. An IMW who went to the emergency room due to a premature rupture of membranes stated:

*“They claimed it was not amniotic fluid, but rather I had urinated on myself. I said I had already given birth to four children. I know the difference between urine and amniotic fluid. They never looked at the amniotic fluid and never performed a cardiotocography. The fluid and blood continued to leak out over the next week.”*

[21]

### 3.3. Forced Reproduction

Many IMW undertake the migratory journey while pregnant; they get pregnant either by their partner or through rape and sexual abuse. As one informant says, networks of traffickers make all decisions related to these women’s SRH, even forcing them to have illegal abortions in Morocco or Algeria: “The network decides if they get pregnant and when or if they will have a child. Sometimes they force them to have abortions in advanced pregnancies” [10]. This problem is compounded by not using contraception, abortions in precarious circumstances, or the use of informal health resources. IMW lack knowledge about sexually transmitted diseases and have little control over their pregnancies, which increases complications in pregnancy, childbirth, or abortions.

#### 3.3.1. Practices That Put the IMW’s Personal Health at Risk

IMWs’ knowledge of sexually transmitted diseases is conditioned by the level of education they received in their country of origin. They have heard of potential risks but do not gauge how serious they are. They sometimes have unprotected sex, even with their partners, and often consent to risky sexual practices out of obligation, sometimes in exchange for protection and food. As one woman says, her partner often refuses to use condoms because he thinks it inhibits sexual pleasure.

*“I asked my partner to use condoms, but he said maleness must be felt and left free. It should not be bound in an envelope (condom). I tried forcing him to use condoms, and he said he would go to his other sweethearts, and I would have to find another partner who uses condoms.”*

[14]

In other cases, IMW sex workers engage in sexual practices that are far from their usual practices, but this is hardly talked about, and they generally do not protect themselves.

*“I do not fuck behind; in my country, we do not talk about it … there is a man with a woman, a woman with a man, but not a woman with a woman, a man with a man, but it is never mentioned …”*

[26]

IMW often do not have access to contraception and complain that it is not provided by healthcare personnel. However, there is also a cultural factor, as they may refuse to use contraception out of tradition. On the contrary, they may use it and hide it from their partners or family, who would not accept such a practice. In other cases, unmarried IMW may have social restrictions on accessing contraception at healthcare centres, so they are unable to prevent unwanted pregnancies. This is what one woman related:

*“My grandmother told me not to have sex before marriage because it is an immoral act. Contraception is only used by unclean women, such as beer girls and prostitutes, before marriage. Clean women only use contraception after marriage; otherwise, their uterus shrinks, making them barren before marriage.”*

[14]

#### 3.3.2. Pregnancies Characterised by IMW’s Irregular Status

Pregnant IMW live in fear of having their irregular status discovered; they seek perinatal care later than others, have fewer home visits, spend fewer days in the hospital, and make fewer visits to healthcare centres. This leads to increased risks during pregnancy and complications in childbirth, such as foetal distress, excessive bleeding, or premature births. In addition, if IMW have not experienced complications in previous births, they do not believe they will have problems with the rest and therefore deal with them in the same way.

*“With my two children, I always started going to the gynaecologist after 6 months of pregnancy. With the other one, I went at eight months, and I had no problems with my son. I said to myself, ‘I can have my daughter without anyone needing to care for me.’”*

[20]

The language barrier is present in the health care they receive throughout the postpartum and childbirth periods, which reduces the quality of care. Due to their culture and origin, IMW are afraid and not aware of all of the pregnancy monitoring methods available. Consequently, they tend to seek fewer obstetric and gynaecological checkups during their pregnancies. This is how one IMW put it:

*“The doctor might check the baby and put the instruments inside the baby, which could accidentally damage it and cause a miscarriage.”*

[21]

Moreover, IMW fear losing their jobs if they become pregnant. They often choose to hide their pregnancies during the first few months and go to healthcare services much later, increasing the potential risks.

*“I got pregnant and was working at the time. I said: ... “The lady will fire me because she does not want me to work.” “Therefore, I did not say anything to the lady.”*

[21]

#### 3.3.3. Unsafe Sex Life

In the face of financial hardship, IMW find themselves forced to have sex with unknown men. They accomplish this as a sign of gratitude or payment for taking care of them, but it is actually prostitution in disguise. These sexual relations are usually unprotected and nonconsensual. Depending on the culture of origin, men may be having sex with several women at the same time and usually do not want to use contraception. This situation increases the risk for IMW:

*“I asked my partner to use condoms, but he said that masculinity should be felt and left free, not tied to a condom. Additionally, he told me that he would leave with his other girlfriends and I should find another partner.”*

[14]

In the case of sex workers, this is a very common situation. They may develop a habit of self-treatment at the first sign of symptoms, an issue that also concerns their partners.

*“No, I do not use protection with my boyfriend’ (sex worker). If it itches, you can use antibiotics or preventative gels...”*

[27]

### 3.4. Alternating between Formal and Informal Healthcare Services

The IMW’s irregular status limits their ability to access public health services. IMW are more afraid of deportation than suffering from a serious illness. Moreover, their beliefs about SRH are influenced by their cultural backgrounds.

#### 3.4.1. Access to Information and Care

To access information about SRH, IMW often seek advice from family, friends, and neighbours. They claim that doctors do not understand them and will not understand their conditions. When faced with SRH problems, going to primary care is not their first option. They lack knowledge and have misconceptions about their health and different conditions. Furthermore, their illegal status is a source of stress for them, as they fear being deported. Barkensjö’s (2018) article shows how access to public health care for IMW is not always easy.

*“They said they could not do anything because I do not have papers,’you are undocumented,’ after sitting there for 10 h.... We felt ignored and drove home.”*

[27]

IMW perceive a severe lack of information, do not feel that their wishes are listened to or respected, and even argue that they may be forced into childbirth at the instruction of healthcare professionals. They generally believe that their opinion is not taken into account by healthcare providers when it comes to making decisions or undergoing risky interventions.

*“No one listened to my wishes. I was forced to have a vaginal delivery, regardless of my pre-existing risks.”*

[28]

IMW also feel that they are not taken seriously and that they are not treated or given the same amount of time as other women.

*“It was really challenging; I was in labour for two days. The doctors came, the interns came, the nurses came; they kept coming, but they did not treat me.”*

[28]

#### 3.4.2. Unsafe Abortions

Unsafe abortions lead to gynaecological complications and severe dangers for IMW. However, the women themselves underestimate the risks of this practice, which are exacerbated by the beliefs, culture, and traditions of their countries of origin. Some IMW claim that contraception causes infertility, so despite the risk, they turn to untrained midwives or take advice from other IMW in similar situations.

*“If I ever notice I miss my period in the first month, I will start clenching and banging my stomach very hard. I will work hard physically; I will jump and massage myself. I will drink a lot of herbal water. If I start early, I will be able to deliver the baby easily.”*

[28]

## 4. Discussion

The aim of this study is to identify qualitative evidence of IMW’s experiences of sexual and reproductive health care in emergency and primary care settings. IMW experience high-risk situations, high rates of physical trauma, extortion, mental illness, and a lack of medical care during the migration process [31,32]. In addition, violence, robbery, sexual harassment, and/or rape make them a very vulnerable group [33]. IMW arriving in Europe in small boats have a history of violence, rape, forced pregnancy, prostitution, and/or trafficking [34]. Regularizing their status in the destination country improves their perceived rights, freedoms, and access to healthcare services.

Emergency care for IMW should include gynaecological examinations and screening for sexual violence and trafficking [10,19]. In primary care, IMW seek consultation on unwanted pregnancies, abortion, family planning, difficulty obtaining contraception, and menstrual irregularities. However, there is a low demand for gynaecological examinations [35,36]. The barriers IMW face in accessing emergency and primary care need to be addressed [32]. Furthermore, healthcare providers do not possess sufficient cultural awareness to adapt their practice [7,18]. For IMW to feel respected, it is fundamental that they know their rights [36]. Positive clinical encounters improve their well-being, peace of mind, empowerment, and trust in healthcare providers; conversely, negative clinical encounters inflict emotional distress and fear [27]. Screening for sexually transmitted diseases and unintended pregnancies is key; healthcare providers can gain the trust of IMW by accompanying them to hospitals in migrant communities and promoting secondary prevention [37]. Physical assessment protocols are needed in emergency care, along with improved coordination between healthcare providers and law enforcement [1,10].

IMW’s concerns about pregnancy, loss of virginity, or sexually transmitted infections need to be addressed. IMW turn to unsafe abortion [14], as they may consider it their only option due to financial constraints or cultural bias. IMW have a higher rate of complications during pregnancy, delivery, and puerperium [21,28], and seek prenatal care later. This is associated with reduced prenatal home visits and obstetric follow-up [38], the use of informal resources due to fear of deportation, job loss, and difficulty accessing public health care [27,28,29]. The division of gender roles consigns women to childcare, while men are in charge of decisions about sex, contraception, and SRH. This would partly explain the frequency of unwanted pregnancies and the use of unsafe contraception [20], thus reflecting the need to involve male partners in caring for IMW [39].

Migrants with infectious or potentially transmissible diseases have a higher perception of stigma [40]. Safe spaces are needed for IMW care in the resettlement phase [41,42]. Many IMW have undergone genital mutilation and need gynaecological checkups [43] but are discouraged by their perception of healthcare providers’ lack of understanding as well as their unempathetic and threatening attitudes [44]. Service provision models should include prenatal and gynaecological screening, language support, well-defined care pathways, community participation, and the involvement of healthcare providers [45]. Screening and follow-up for sexually transmitted diseases are critical [46]. Young IMW and sex workers face barriers to accessing SRH services [47]. Studies show associations between irregular migration, sexually transmitted diseases [14], and insufficient contraception [20]. Sex workers are aware of STD risks with clients but not with their partners [26]. They also refuse to use contraception to avoid infertility, resulting in unwanted pregnancies and unsafe abortions [14,27].

IMW have social expectations, cultural norms, and a limited understanding of SRH [14]. Their socioeconomic level in their country of origin is related to making responsible choices [1,26]. These women tend to look for information from their peers or from religious institutions, whereas men prefer to look on the internet. Institutions often do not have information and are not familiar with the system [1,21,29]. The situation also differs depending on the country; it is easier for IMW to access SRH services in Spain than it is in Switzerland or Lebanon [21,28].

Healthcare providers see an abundance of mental health conditions, a lack of social support, fear, and psychological trauma among IMW. Postraumatic stress, depression, and anxiety increase the risk of postpartum depression, hence the need to include mental health screening in SRH care [1,13,21,27]. Fear of deportation is also an obstacle for IMW seeking medical care; in countries such as Sweden, clearer guidelines for healthcare professionals and IMW are needed [48]. Strategies for improving IMW’s access to care include training professionals, giving IMW access to community volunteers and cultural mediators, and creating an open-door culture for accessing health care [49]. There is also a need to break down language barriers so that IMW can make medical appointments and avoid misunderstandings [10,21,27,29]. Some studies argue that the use of interpreters leads to a lack of confidentiality [21]. Nonetheless, this issue needs to be addressed as the language barrier causes stress and misunderstandings, resulting in IMW feeling distrustful, discriminated against, and discouraged from returning to formal healthcare services [2,29].

## 5. Conclusions

The harsh migration journey increases the vulnerability of IMW, who are victims of physical, psychological, and sexual violence [50,51]. Safety protocols and emergency/primary care focused on SRH need to be established for IMW. Care includes physical, obstetric, and gynaecological assessments, as well as the detection of sexual violence and human trafficking. IMW’s lack of information increases their perception of risk as they feel deceived and ignored. IMW find themselves forced to have children due to gender issues; men are in control of decisions surrounding reproduction, and IMW are often victims of rape and human trafficking. IMW’s irregular status can have a negative impact on pregnancy and the associated health checks. When IMW go to healthcare centres regarding STDs or pregnancy, they feel afraid, insecure, neglected, stigmatised, and uninformed. Cultural aspects and fear of deportation can be linked to IMW not seeking care and undergoing unsafe abortions. Healthcare providers lack awareness and training surrounding care for IMW. In order to provide competent care, they require specific training in SRH.

## 6. Implications for Practice

Public institutions should implement specific protocols for providing SRH care to IMW in emergency and primary care. They should cover safety and the detection of covert prostitution and human trafficking. Healthcare providers need specific training in SRH care for IMW, including cultural competencies of active listening, communication, trust, and the promotion of women’s autonomy. IMW use health services less frequently and later. They are also more likely to withdraw from SRH treatment and programmes. Therefore, active follow-up programmes by healthcare providers need to be developed. Social, cultural, or religious issues may increase the risk of sexually transmitted diseases, unwanted pregnancies, and unsafe abortions. These need to be taken into account in primary care and emergency consultations.

## Figures and Tables

**Table 1:** Strategies of search.

Stage	Steps
STAGE 1	The terms “illegal,” “irregular,” and “undocumented” were joined with the Boolean operator “OR.” This process was carried out with “migrant,” “immigrant,” “foreigners,” and “noncitizen,” as well as with “pregnancy,” “sexual health,” “maternal health,” “health care,” “reproductive health,” “qualitative research,” and “women.”
STAGE 2	After performing these searches separately, they were joined together using the Boolean operator “AND.” (((((((migrant) OR immigrant) OR foreigner)) AND (((((undocumented) OR illegal) OR irregular)) OR noncitizen)) AND ((((((pregnancy) OR sexual health) OR maternal health) OR health care) OR reproductive health) OR health services)) AND ((women) OR human female)) AND ((qualitative research) OR qualitative design).
STAGE 3	In addition to electronic searches, a manual search of grey literature was carried out.

**Table 2:** Evaluation of the quality of the studies [25].

Article	1	2	3	4	5	6	7	8	9	10
[14]	✔	✔	✔	✔	✔	↔	✔	✔	✔	✔
[1]	✔	✔	✔	✔	✔	✔	✔	✔	✔	✔
[26]	✔	✔	✔	✔	✔	↔	↔	✔	✔	✔
[20]	✔	✔	✔	✔	✔	↔	↔	✔	↔	✔
[21]	✔	✔	✔	✔	✔	✔	✔	✔	✔	✔
[10]	✔	✔	✔	✔	✔	↔	✔	✔	✔	✔
[27]	✔	✔	✔	✔	✔	↔	↔	✔	✔	✔
[28]	✔	✔	✔	✔	✔	↔	✔	✔	✔	✔
[29]	✔	✔	✔	✔	✔	↔	✔	✔	✔	✔

NOTE: ✔ Yes, **↔** Unclear. 1. Congruence of philosophical perspective/methodology; 2. Congruence of methodology/objectives; 3. Congruence of methodology/data collection; 4. Congruence of methodology/data analysis; 5. Congruence of methodology/interpretation of results; 6. Cultural and theoretical context of the researcher; 7. Influence of the researcher on the research; 8. Participants represented; 9. Research Ethics Committee Approval; 10. Conclusions from data analysis/interpretation.

**Table 3:** Stages in the thematic synthesis process [30].

Stage	Description	Steps
STAGE 1	Text coding	Recall review question Read/re-read the findings of the studies Line-by-line inductive coding Review of codes in relation to the text
STAGE 2	Development of descriptive themes	Search for similarities/differences between codes Inductive generation of new codes Write preliminary and final report
STAGE 3	Development of analytical themes	Inductive analysis of sub-themes Individual/independent analysis Pooling and group review

**Table 4:** Characteristics of the chosen studies.

Author and Year	Country	Sample (IMW)	Age (Years)	Interview Duration	Data Collection	Data Analysis	Main Theme
[14]	Cambodia	15	18–28	Not interviewed	IDI	Manual analysis of coded data	Attitudes toward or practise of unsafe abortions
[26]	Spain	8	23–40	30 min	IDI	Content analysis	Risk of STIs and HIV in sex workers
[20]	Spain	26	20–35	3 h	FGs	Thematic analysis	IMW’s experiences of maternity care
[21]	Switzerland	33	21–40	Not interviewed	FGs	Analysis of themes and subthemes	Experiences of maternal health services
[10]	Spain	13	18–35	18 min	IDI	Valerie Fleming stages	IMW’s health needs
[27]	Sweden	13	18–36	45 min	IDI	Qualitative analysis of content	Clinical experiences of birth/pregnancy
[28]	Lebanon	35	Not provided	1 h	IDI	Ethnographic analysis of themes	Unequal access to care for IMW
[29]	United States	8	20–45	Not interviewed	Life story	Analysis of statements	Cultural needs and access restrictions
[1]	Belgium Netherlands	14	15–49	Not interviewed	IDI	Inductive analysis	Sexual health determinants

IMW = irregular migrant women. IDI = in-depth interview. FGs = focus groups.

**Table 5:** Themes and subthemes.

Themes	Subthemes
3.1. The need to focus emergency care on SRH	3.1.1. IMW: victims of trafficking and sexual exploitation.
	3.1.2. The need to develop suitable safety protocols
3.2. Unsatisfactory clinical experiences	3.2.1. The need for interpreters.
	3.2.2. Healthcare providers’ lack of cultural competence
3.3. Forced reproduction	3.3.1. Practices that put the IMW’s personal health at risk
	3.3.2. Pregnancies characterised by the IMW’s irregular status
	3.3.3. Unsafe sex life
3.4. Alternating between formal and informal healthcare services.	3.4.1. Access to information and care
	3.4.2. Unsafe abortions

SRH = Sexual and Reproductive Health. IMW = Irregular Migrant Women.

## Data Availability

The data presented in this study are available on request from the corresponding author.
